# Supraventricular Arrhythmias after Thoracotomy: Is There a Role for Autonomic Imbalance?

**DOI:** 10.1155/2013/413985

**Published:** 2013-10-23

**Authors:** George Vretzakis, Marina Simeoforidou, Konstantinos Stamoulis, Metaxia Bareka

**Affiliations:** Anesthesiology Clinic, University Hospital of Larissa, 41110 Mezourlo, Greece

## Abstract

Supraventricular arrhythmias are common rhythm disturbances following pulmonary surgery. The overall incidence varies between 3.2% and 30% in the literature, while atrial fibrillation is the most common form. These arrhythmias usually have an uneventful clinical course and revert to normal sinus rhythm, usually before patent's discharge from hospital. Their importance lies in the immediate hemodynamic consequences, the potential for systemic embolization and the consequent long-term need for prophylactic drug administration, and the increased cost of hospitalization. Their incidence is probably related to the magnitude of the performed operative procedure, occurring more frequently after pneumonectomy than after lobectomy. Investigators believe that surgical factors (irritation of the atria per se or on the ground of chronic inflammation of aged atria), direct injury to the anatomic structure of the autonomic nervous system in the thoracic cavity, and postthoracotomy pain may contribute independently or in association with each other to the development of these arrhythmias. This review discusses currently available information about the potential mechanisms and risk factors for these rhythm disturbances. The discussion is in particular focused on the role of postoperative pain and its relation to the autonomic imbalance, in an attempt to avoid or minimize discomfort with proper analgesia utilization.

## 1. Introduction

Variations from the normal rhythm of the heartbeat, encompassing abnormalities of rate, regularity, site of impulse origin, and sequence of activation are well-documented complications following thoracotomies for surgical treatment of intrathoracic pathology [1–10]. Generally, these perioperative arrhythmias are associated with longer hospital stay and higher cost [8]. Although not widely accepted, many authors believe that patients developing persistent or recurrent tachyarrhythmias present a greater mortality and experience more complications than those who maintained normal sinus rhythms after surgery [1–3]. Based on an up-to-date literature review, this paper discusses currently available information about the potential mechanisms and risk factors for the development of these rhythm disturbances.

We searched for original articles, review articles, case series or case reports, and editorials using the MEDLINE database (from January 2002 to September 2012) and the Cochrane Central Register of Controlled Trials by combining the terms “thoracotomy,” “pulmonary resection,” “arrhythmia,” “atrial fibrillation,” “heart rate variability,” and “pain.” The search was limited to humans and adults and only publications with at least an English abstract were included. Publications without abstracts were excluded, except from case series and case reports. MEDLINE database was searched using PubMed advanced search with the following strategies: (1) *thoracotomy AND arrhythmia*, 149 results; (2) *pulmonary resection AND arrhythmia*, 86 results; (3) *atrial fibrillation AND pulmonary resection*, 76 results; (4) *heart rate variability AND thoracotomy AND arrhythmia*, 1 result; (5) *thoracotomy AND pain AND arrhythmia*, 12 results. All identified citations that seemed relevant to our scope were reviewed. Some citations were generated in common by the abovementioned strategies. Initial consideration excluded citations not focusing on the potential mechanisms for development of supraventricular arrhythmias after thoracotomy. We also excluded citations dealing with preventive measures and techniques and drug therapies. This search strategy yielded 32 articles. We expanded inclusion to the reference lists of these articles, and finally 89 articles were found to be of relevance. The full texts of these original articles, review articles, case series, case reports, and editorials were retrieved and examined. Finally, 84 articles were included in this narrative review.

A number of factors have been identified as related to higher risk for the appearance of postthoracotomy arrhythmias. In the literature, factors considered in favor for the development of supraventricular arrhythmias are advanced age, male sex, history of chronic obstructive pulmonary disease (COPD), major resection especially right-sided, mediastinal dissection, and intrapericardial pneumonectomy. In individual patients, preexisting cardiac disease, hypoxemia, hypercapnia, intraoperative fluid imbalance, hypotension, and other factors may also be related to the risk of arrhythmia.

Little is known about the potential mechanisms that generate these arrhythmias. As it will be discussed in the following, some data exist that it is inflammation in aged atria that predisposes to arrhythmia in case of surgical irritation or damage. The correlation of ectopic rhythms with hypoxia, hypercapnia, acidosis, and hyperphosphatemia is also noted by many researchers. As most of the studies analyzing the risk factors were retrospectively performed and the effectiveness of perioperative analgesia could not be revealed, very little is known directly and definitively for the role of pain. Postthoracotomy pain deserves extensively commenting for, at least, five reasons. Firstly, it is significant and sometimes intolerable if untreated. A second reason is that the onset of arrhythmias parallels with pain. A third reason is that analgesic treatment with epidurally administered local anesthetics may result in significant decrease in the incidence of arrhythmias. Another reason comes from the fact that pain may cause significant cardiac autonomic imbalance predisposing to arrhythmia due to increased sympathetic outflow. A fifth reason favoring a relation of pain and autonomic imbalance to postthoracotomy arrhythmias is the observation that, in most of the cases, arrhythmias show good clinical course and recurrence of normal sinus rhythm as pain decreases. Although the impact of other analgesic modalities as the intravenous administration of opioids on the autonomic system is different, information about their prophylactic role is missing. Finally, the role of the direct irritation or injury to the anatomic structures of the autonomic system is suggested by a number of investigators [10], but the information on this subject is scarce.

## 2. Incidence

The major rhythm disturbances after operations on pulmonary parenchyma, for malignancy or not, or other intrathoracic structures are atrial fibrillation (AF) (most commonly), supraventricular tachycardia (SVT), and atrial flutter. Most of the studies do not include patients with premature ventricular complexes or atrial extrasystoles. Ventricular tachycardias were extremely uncommon in the studies reviewed.

Supraventricular arrhythmias (SA) may be seen throughout the whole perioperative period in these operations. An overall incidence of 3.27% was reported for intraoperative AF in a retrospective review of 10563 medical records of patients who underwent lung operations [11]. In general, the reported incidence of postoperative SA is higher, varying from 8.3 to 31.9% [1–10, 12, 13], peaking on the second [9, 13] or third postoperative day [3]. In a prospective database including 2588 patients the incidence of AF was 12.3% [14]. The percentage of patients developing postoperative arrhythmia is higher after pneumonectomy compared to that after lobectomy. Bilobectomy is often listed with pneumonectomy. The incidence of tachyarrhythmias in nonanatomic resections (wedge resection) may range from almost null to approximately 10% and is very low also in segmentectomies [7, 10]. Esophagectomy is also followed by rhythm disturbances [14–16]. In a retrospective analysis of a period greater than 10 years including 921 esophagectomies for cancer the incidence of AF was 22% [17]. Hence, the overall incidence varies between series, depending on the percentages of the different types of operations and on the particular types of arrhythmias which were included for the calculations.

## 3. Risk Factors

In older reports, gender and advanced age were not reported as important predisposing factors for tachyarrhythmias after pulmonary resections [2, 5, 7]. The independent role of these two factors was obscured by the different incidence of concomitant cardiopulmonary diseases. Male gender and older age are often correlated with a longer smoking history and COPD and thus with a higher probability of impaired oxygenation and cardiac dysfunction. Nevertheless, smoking history could not be identified as a predictor of AF in both pneumonectomy and lobectomy patients [10]. More recently, male sex and advanced age were documented as strong clinical predictors for the appearance of SVT [3, 4, 10, 13, 18, 19]. Aging causes degenerative and inflammatory changes in atrial myocardium and, consequently, it leads to alterations in the electrical properties of the atria [20, 21]. In addition, in an experimental setting, older animals developed significantly more episodes of supraventricular arrhythmias after pneumonectomy than their younger counterparts [22]. It has been reported that age >60 years was the only independent preoperative risk factor most strongly and consistently associated with postoperative AF [23].

Preoperative function tests were repeatedly evaluated and presented no significant predictive value for the prediction of postoperative cardiac function and hemodynamic disturbances [2, 5, 23, 24]. Many authors agreed that there was no apparent association with standard preoperative pulmonary function tests and the incidence of postoperative arrhythmia. In two retrospective analyses, FEV_1_ (cut-off point of 50% [5] or 2.0 L [2]) failed to affect significantly the prevalence of AF. Nevertheless, the concept of using arbitrary cut-off values for categorizing patients in clinical studies has many limitations. It was recently shown that patients suffering from COPD develop arrhythmias in the postoperative period significantly more often than no COPD patients do. In a retrospective analysis of 244 patients, the incidence of SVT and AF differed significantly between COPD (FEV_1_ 1.5 L, 48.37%) and non-COPD patients (FEV_1_ 2.3 L, 79.6%) [9, 19]. Furthermore, the history of chronic lung disease was found independently predictive of SVT in a series of 4000 patients [25].

Conflicting data exist about the role of preoperative hypoxemia on the incidence of postoperative supraventricular arrhythmias. It is known from past research that animals that were made hypoxic developed arrhythmias and had hemorrhage of the conducting system [26] and that ectopic rhythms correlate with hypoxia during operation [27]. Hypoxia is known to cause changes in the electrical characteristics of cardiac muscle that can lead to arrhythmias [28]. Nevertheless, a retrospective study showed that SpO_2_ values less than 90% were not significantly associated to SVTs [5]. More recently, PaO_2_ values showed no difference between patients with (PaO_2_ = 78.5 mm Hg) and without (PaO_2_ = 80.7 mm Hg) postoperative AF [10]. In disagreement with the above, other series documented significantly lower preoperative values of PaO_2_ in patients who developed tachyarrhythmias [7, 9]. Postoperatively, at the time of onset of this complication, patients are not necessarily in a condition of severe hypoxemia [2, 7].

Most authors agree that the incidence of tachyarrhythmias is related to the magnitude of the operative procedure performed, occurring more frequently after pneumonectomy than after lobectomy [2, 4, 5, 7, 10, 12, 29] (Table 1). Major resections are independently predictive of postoperative arrhythmias and perioperative mortality [9]. In a prospective study with a small study population, patients developed postoperative arrhythmias at almost equal percentages [16]. The development of postoperative rhythm disturbances is not associated with the particular indication of surgery (malignancy or not) and the underlying pathology. In 298 patients, benign tumors, tuberculosis, and other lung diseases represented the 27% of the indications for surgery. These indications did not correlate significantly with the development of postoperative arrhythmias [5]. Moreover, in case of malignancy, the stage of lung cancer is also not associated with the incidence of arrhythmia. Lung cancer, not because of its malignancy, but because of its association with a history of smoking and COPD, or because it requires a more extensive lung resection, is sometimes considered as a risk factor for the onset of postoperative arrhythmia [6].

Tachyarrhythmias occur more frequently in operations with intrapericardial dissections or pericardial handling and in cases of postoperative interstitial or perihilar pulmonary edema [2, 3, 24, 30]. The operational side may also play a role in the occurrence of postoperative arrhythmias. Many authors, comparing right and left pneumonectomies, demonstrated a higher incidence after right-sided procedures [3, 13]. Reported results from other investigators do not confirm this observation [10]. Postoperative hemorrhage and repeat thoracotomy are also significantly associated with AF [5]. Finally, a relationship was suggested between previous thoracic irradiation and the frequency of postthoracotomy cardiac rhythm disturbances [31].

## 4. Potential Mechanisms

Increased atrial ectopy, atrial premature contractions, and acutely increased heart rate may lead to reentry of multiple small wavelets and initiate a paroxysm of atrioventricular nodal tachycardia and/or AF [32]. It has been observed that a greater preoperative heart rate (HR) was an independent predictor of AF after cardiac operations, especially among the elderly [22]. Atrial fibrosis or inflammation per se or after an indirect atrial trauma during surgery presumably provides the abnormal milieu for the development of SVT, especially in the elderly [33] and during operations implicating major resections, interpericardial dissections, and pericardial handling. Experimental information is supportive for a relation between aging, inflammation, and postpneumonectomy supraventricular arrhythmias [34]. In the past, atrial inflammation was included among the proposed causes of arrhythmia by some authors [2]. Up to now, the data about the role of inflammatory changes on the appearance of post-thoracotomy tachyarrhythmias remain extremely scarce. Acute pericarditis was considered a primary initiator of AF after cardiac operations, but this has never been proven [35].

The role of direct injury to the autonomic cardiac innervation during thoracotomies is speculated by a number of reports. The heart is innervated by both sympathetic and parasympathetic filaments, accompanying the coronary arteries and their branches. Autonomic cardiac nerves may be exposed, retracted, and injured during thoracic surgery. Injury to the parasympathetic branches of the sinoatrial node may result in increased heart rate and decreased atrioventricular conduction. Surgically induced alterations in the efferent sympathetic outflow to the heart may account for postoperative cardiac arrhythmias. The anatomic location of cardiac plexus in the area between the aortic arch and the tracheal bifurcation makes it vulnerable to direct damage, especially in thoracotomies implicating dissection of pulmonary hilum or sampling of nodes located in this area. Meanwhile, there is no data concerning possible impulse generation at the site of surgical incision by the application of surgical instrumentation, pressing and deforming thoracic wall structures, and by the partial removal of costa. It is unknown if such impulses reach sympathetic ganglia or more central anatomic formations and return to the heart as an altered autonomic tone. Recently, in patients undergoing sympathectomy for primary hyperhidrosis, with a minimally invasive endoscopic technique, decreased HR was observed postoperatively [36]. This clinical vagotonic image of the heart was further documented as a decrease in sympathetic activity by heart rate variability (HRV) analysis. In another report, patients who underwent video-assisted thoracoscopic lobectomy for lung cancer were properly matched with those undergoing open thoracotomy and postoperative atrial fibrillation developed in comparable percentage of patients in both groups [37]. This supports the theory that autonomic denervation may be responsible for the pathogenesis of postoperative SA. Finally, other anatomic regions may also play a role. It has been shown that pulmonary vasculature proximally to the heart shows electrical properties which can generate ectopic beats [38]. Surgical irritation or damage to this zone may probably be arrhythmogenic.

The observation that a greater preoperative heart rate is an independent predictor of postoperative AF suggests a relation of vagal tone with the appearance of these arrhythmias. In fact, some believe that lower vagal tone stratifies patients susceptible to AF [22]. Autonomic imbalance has been implicated as a possible trigger of postoperative AF. Nevertheless, increased heart rate may also be attributed to increased sympathetic drive to the heart, and there is some controversy as to whether the imbalance in relation to the postthoracotomy arrhythmias is primarily vagal or sympathetic in origin [39, 40]. As it has been shown in experimental models, AF may be produced from increased vagal tone alone. Some hypothesize that the heightened background sympathetic tone helps promote AF with increased vagal activity. It is conceivable that vagal rebound promotes ectopy by shortening refractoriness in a heterogeneous matter and that the increased background sympathetic activity may then promote AF [41]. The adrenergically or vagally mediated AF in nonsurgical patients, which, in respect, is characterized by an increasing or decreasing HR before arrhythmia onset, may be interpreted differently in postoperative patients. A complex postoperative environment that consists of changing respiratory pattern, ensuing pain, application of various analgesic techniques, including central neural blockade, and the systemic circulation of swinging opioid concentrations could possibly affect the arrhythmogenicity potential in the individual patient.

It is known that during normal sinus rhythm the time interval between heartbeats is not stable. This beat-to-beat oscillation of RR intervals around its mean value is heart rate variability. Measured and analyzed, this physiological phenomenon of cycle length variability represents a simple and noninvasive electrocardiographic quantitative index of the heart's autonomous status [42–45]. A typical spectrum of RR series is characterized by two main frequency components, namely, the high-frequency component of the power spectrum (HF) and low (LF). In general the HF component has been thought to reflect primarily vagal activity whereas LF reflects both sympathetic and vagal activity. The ratio of LF to HF power is often considered a measure of sympathovagal balance. Nevertheless, HRV is only an indirect measure of autonomic influences on the sinus node, and therefore interpretation concerning the exact mechanism of postoperative AF should be made with caution [46].

Denervated hearts show typically higher heart rates than innervated hearts and much reduced HRV with no definite spectral components [47, 48]. The abovementioned postthoracotomy tachycardia, which is associated with decrements in all HRV variables, could also be explained by “apraxic surgical injuries” to both sympathetic and parasympathetic neural fibres, leading to a temporary state of denervated heart.

Is an imbalance of heart's autonomic system somehow present during thoracotomy? Findings about heart's autonomous nervous system during esophagectomy or pulmonary resections were demonstrated in patients operated without epidural anesthetic support [16]. After esophagectomy LF, HF, and total-frequency components of HRV were reduced, LF/HF ratio was increased. In the pulmonary resection group, declined LF component of HRV, reduction of the total-frequency component of HRV, decreased LF/HF ratio, and no significant change in HF were observed. These results indicate decreased sympathetic outflow to the heart. Postoperatively, these patients presented increased heart rates, irrespective of the performed operation and supraventricular arrhythmias that occurred in 15% of them. More recently, decreased LF component and increased LF/HF ratio were demonstrated in patients undergoing thoracic surgery under high thoracic epidural analgesia (TEA) [49]. These results also indicate decreased sympathetic activity, but attributing it solely to the performed thoracotomy is incorrect as TEA also may lead to similar HRV findings (see below). Using HRV analysis, other investigators also showed that thoracoscopic sympathectomy under general anesthesia is followed by blunted cardiac sympathetic neural drive which is in agreement with what is clinically expected. Postoperatively, HR was decreased, and the 38 patients of this study did not developarrhythmias [36].

In a prospective study, Holter recordings were compared between patients with postthoracotomy AF and matched surgical controls without AF [50]. Patients developing AF had a significant elevation of heart rate before developing arrhythmia. The authors analyzed HRV data before the onset of AF and found that both LF and HF powers were significantly greater than those in the nonarrhythmia periods. They also had significantly lower LF/HF ratio compared to controls. These data suggest that vagal activation in the setting of sympathetic predominance contributes strongly to postoperative AF. This study differs in comparison to the abovementioned studies, as it does not only demonstrate the changes in autonomic status after thoracic surgery, when there is an increased possibility for arrhythmia development, but it also focuses in detail on the autonomic alterations that precede the onset of arrhythmia. The findings of this study are comparable to data in nonsurgical patients where RR intervals decreased and frequency domain parameters of HRV increased before AF onset [51]. In addition, time domain parameters of HRV also increased in the study patients, providing consistent data to suggest that vagal rebound or resurgence was occurring in addition to an elevation in sympathetic tone before the initiation of postoperative AF.

Thoracotomies are followed by significant pain. Thoracic epidural anesthesia is regarded as the most reliable treatment for postthoracotomy pain management [52, 53]. TEA with administration of local anesthetics shows a prophylactic effect on postoperative dysrhythmias after thoracotomy [8, 54, 55]. These observations are somehow indicative of a relationship between postthoracotomy pain and the appearance of arrhythmias during the postoperative period. Increased sympathetic outflow due to postoperative pain may be arrhythmogenic. Most authors have verified that TEA's analgesic effect associates with decreased sympathetic drive to the heart. For thoracotomies, high TEA (sensory block of C6-T6) is preferable as it is associated with minimal effect on systemic vascular resistance and more or less stable hemodynamics [56, 57].

It is known that high TEA does not alter the overall reflex responses to a number of stimuli and is associated with stable hemodynamics and preservation of baroreflex sensitivity. In a study incorporating HRV analysis, the decreased sympathetic outflow after TEA was associated with some withdrawal of vagal activity, as LF/HF ratio was increased [57]. In the same patients, induction to general anesthesia, laryngoscopy, and tracheal intubation was also followed by increased LF/HF ratio and the authors attributed this change to sympathetic activation. In another study, with sensory block of C6-T6, both low- and high-frequency spectral power remained unchanged. During tilting, the subjects showed attenuation of the increase in heart rate, no change in absolute and fractional LF and HF power, and a decrease in LF/HF ratio which was attributed to preganglionic cardiac sympathetic blockade [58]. The authors' interpretation of these findings was subsequently criticized in the literature [59]. Finally, the combined effect of thoracotomy and TEA on HRV variables was demonstrated, suggesting autonomic imbalance and decreased postoperative sympathetic outflow [49]. Decreased HF and LF components and decreased LF/HF ratio were demonstrated early after surgery, with total HRV and HF increasing towards preoperative values whereas the LF/HF ratio remained significantly lower.

More studies are needed to elucidate the role of analgesic therapies on the prophylaxis of postthoracotomy arrhythmias. Effective, clinically adequate analgesia does not preclude a pro- or antiarrhythmic effect. Although opioids can increase parasympathetic activity and may balance heightened sympathetic tone after operation, they exhibit no effect on sympathetic drive. The effect of opioids on HRV variables is different than that of the epidurally administered local anesthetics [60]. Postoperative patient-controlled analgesia (PCA) with opioids was compared with TEA after thoracic surgery under general or combined general/epidural anesthesia [61]. In this study, HRV analysis was not used. The incidence of SVT, AF, and supraventricular ectopic beats was lower in the opioid group (PCA). These results are in disagreement with the findings of a similar study where postoperative tachycardia was significantly less frequent in the TEA group [49]. In this later study, HRV analysis showed unchanged LF/HF ratio in the opioid-PCA group whereas it was significantly reduced in the TEA group. Similarly, in patients undergoing thoracotomy under combined general and thoracic epidural analgesia with levobupivacaine, it was found that substitution of the local anesthetic on the 3rd postoperative day with patient-controlled intravenous morphine resulted in an increased LF component of the HRV [62]. Patients in the continued TEA group were free of postoperative arrhythmias. This difference in sympathetic outflow was not clinically apparent as there was no difference between the groups (TEA with levobupivacaine versus morphine PCA in the 3rd day) regarding hemodynamic variables and pain visual scale. The authors concluded that the postoperatively decreased cardiac sympathetic outflow continues with epidural local anesthetics administration, whereas it is abolished by the change to intravenous patient-controlled morphine. Finally, two studies tested the impact of TEA on postoperative AF in patients who underwent coronary revascularization [63, 64]. In both, TEA showed no effect. The lack of prophylaxis in the second study existed despite a significant reduction in sympathetic activity assessed with HRV analysis. It seems that the underlying arrhythmogenic mechanisms differ significantly between thoracic and cardiac operations.

## 5. Clinical Significance

Most commonly, the onset of supraventricular arrhythmias follows the operation, not rarely complicating the period when patients regain consciousness. Intraoperative incidence compared to postoperative is considerably lower. The course of these arrhythmias varies. In the majority of cases, patients leave the hospital in normal sinus rhythm or, in lower percentages, they are dismissed arrhythmic but with good rate control under medication. However, a significant percentage of patients undergoing thoracic surgery die in the hospital, and sometimes it is difficult to clear among other factors the role of arrhythmias for this outcome. Complications such as atelectasis, pneumothorax, pneumonia, bronchopleural fistula, empyema, and acute respiratory distress syndrome are not uncommon in the postoperative period, especially in patients with history of COPD [9]. Recurrence of supraventricular arrhythmia may be encountered when, in the course of postoperative hospitalization, a respiratory complication occurs [13, 65]. The importance of the postthoracotomy arrhythmias lies in the immediate hemodynamic consequences, the potential for systemic embolization and the consequent long-term need for prophylactic drug administration, and the increased cost of hospitalization. In 28% of “arrhythmic” patients elevation of concentrations of cardiac enzymes was found, with or without electrocardiographic signs of myocardial ischemia or infarction, and in 38% hypotension was observed [2]. Even when atrial fibrillation is a solitary complication, hospital stay for these patients is longer [13]. Many investigators have stated that the patients who develop persistent or recurrent tachyarrhythmias present a greater mortality and experience more complications with longer hospitalization and higher costs than those who maintained normal sinus rhythms after surgery [1–3, 8, 13].

Prevention and treatment of postthoracotomy arrhythmias is an important issue for the clinician. Nowadays, the acceptance and wide postoperative use of analgesic techniques with local anesthetics or opioids make it somehow unethical to perform prospective studies investigating the impact of pain on their onset and clinical significance. There are no studies directly relating pain, irrespectively, if it exists intraoperatively in a hypnotized patient, during emerge, or in a fully awaken patient postoperatively, with the appearance of arrhythmias. However, the “antiarrhythmic” properties of a perioperative pain-free status are shown in many of the aforementioned studies [8, 54, 62]. Furthermore, data in these reports show that high TEA is more prophylactic than intravenous opioids.

Until a few years ago, the effectiveness of antiarrhythmic prophylaxis with drugs in the course of thoracic operations was debatable [10, 55]. Some are keen to reverse early atrial fibrillation by electrical defibrillation ([2], see also Appendix A in [5]). Although this review is not focused on the preventive drug therapies, some results from the reviewed literature are provided here and they are summarized in Table 2. Existing reports vary in design, size of studied population, and quality of data. In the past, digoxin was found ineffective for the prevention of AF in patients undergoing a variety of thoracic surgery procedures [66–68]. Two small studies showed better results for flecainide and diltiazem compared to digoxin [69, 70]. Flecainide versus placebo was found effective for the prevention of AF with no side effects [71]. In a relatively strong randomized study of 330 patients, the prophylactic effect of diltiazem was evaluated [18]. The study demonstrated a significant reduction in postoperative AF, with an incidence of 26% in control patients which was reduced to 14% in diltiazem patients, without significantly increased adverse reactions to drug treatment. Beyond atrial arrhythmias, calcium channel blockade is associated with a significant reduction in the incidence of the not very common in these operations ventricular arrhythmias [72].

In a three-arm trial of verapamil, amiodarone, and controls, the first drug reduced the incidence of AF but this finding was lacking significance and, at the same time, a significant proportion of patients experienced bradycardia or hypotension. The amiodarone arm was stopped early after three patients developed ARDS and two of them died of this complication [73, 74]. However, the dose of amiodarone in these patients was relatively high (1200 mg/24 h being given i.v. for 3 days). In a more recent but retrospective and small study of 83 thoracic surgery patients, the incidence of AF in the amiodarone group was 10%, significantly lower than controls (33%) with no complications for the drug [75]. The dose of amiodarone was far lower (200 mg orally) in this study. Amiodarone effectively decreased the incidence of postoperative arrhythmias in a number of other trials without serious side effects [7, 12, 65]. In a randomized study of 130 thoracic surgery patients, a continuous infusion dose of 1050 mg of amiodarone, given after anesthesia induction for the next 24 hours, followed by oral administration of 800 mg for a maximum of 6 days, was proven effective for reducing the incidence of AF significantly [76]. The incidence of pulmonary complications or other adverse effects was not different compared to controls, and, moreover, patients belonging to the amiodarone group left intensive care unit earlier. More recently, in a randomized controlled study of 254 patients, 300 mg of amiodarone was given intravenously over 20 minutes immediately after surgery, and it was followed by an oral dose of 600 mg twice daily during the first 5 postoperative days. This scheme lowered significantly the incidence of AF without increasing complications [77]. In case that cardioversion is decided, for example, when AF persists and is followed by hemodynamic consequences, electrotherapy under amiodarone has more possibilities for conversion and preservation of sinus rhythm [78]. However, existing reviews underline the fact that the appropriateness of amiodarone prophylaxis against postthoracotomy AF is not the same as in the course of cardiac surgery [79].

Other prophylactic strategies include the administration of beta-blockers (metoprolol and propranolol). However, studies for antiarrhythmic prophylaxis with these drugs suffer from small-sized populations, not clearly significant results, and that the patients experienced side effects [80, 81]. Existing reviews support the use of beta-blockers after individualization and with having the possible adverse events in mind. Finally, as a link has been established between low magnesium plasma concentration and the generation of arrhythmias, a prophylactic magnesium treatment has also been proposed [82–84].

## 6. Conclusions

Thoracic surgical interventions involving excision of lung tissue are known to be associated with various forms of supraventricular arrhythmias, most commonly AF. The authors dealing with the subject have revealed certain predisposing-precipitating factors, such as age, male gender, pneumonectomy (especially right), bilobectomy, intrapericardial procedures, and COPD. As to the pathophysiological background of post-thoracotomy tachyarrhythmias, there is still much to be explained. Autonomic nervous system disorders triggered both by intraoperative surgical manipulations as well as by potent pain stimuli, seem to be of cardinal importance for the genesis of the aforementioned arrhythmias; the latter also explains the potential beneficial role of TEA in regard to prophylaxis against tachyarrhythmias. Heart rate variability consists of a useful, noninvasive diagnostic tool that has been recruited in order to elucidate various aspects of the related pathophysiology, showing that postoperative tachyarrhythmias can partly be attributed to a perioperative disequilibrium between the basic variants of the autonomic system—the vagal and sympathetic ones. In regard to the prophylactic/therapeutic approach and in addition to the favourable effect of TEA, most authors agree that the application of pharmacological agents such as amiodarone and beta or calcium channel blockers can either prevent or suppress tachyarrhythmias with high rates of success. Finally, the maintenance of a physiologic electrolytic balance with prompt correction of related deviations, especially the ones affecting potassium and magnesium serum levels, is another way to protect our patients from this type of postthoracotomy complications, which can cost lives if not dealt with properly.

## Figures and Tables

**Table 1:** Association between postoperative heart rate disorders and the type of the performed surgical procedure.

Study	Ref. no.	Included arrhythmias	Percentage in pneumonectomy*	Percentage in lobectomy	Overall
Krowka et al. 1987	[2]	TDR	23.6**		
Dyszkiewicz and Skrzypczak 1998	[5]	AF	24.0	6.1	8.3
Curtis et al. 1998	[6]	SA	39.4	17.0	22.4
Ciriaco et al. 2000	[7]	SA	33.3	12.6	13.7
Sekine et al. 2001	[9]	SVT			31.9
Rena et al. 2001	[10]	SA	35.0	23.0	22.8
Barbetakis and Vassiliadis 2004	[12]	SA	37.3	17.4	21.6

*Including bilobectomy.

**Study addressing only pneumonectomy.

TDR denotes tachydysrhythmia.

**Table 2:** Drug prophylaxis for postthoracotomy arrhythmias.

Drug	Study (ref. no.)	Study type	Number of patients	Results	Study strength/weakness
Digoxin (see also studies [69, 70])	Ritchie et al. 1990 [66]	Unblinded PRCT	Digoxin, *n* = 64 Control, *n* = 66	Incidence of arrhythmia Digoxin 29/64 (45%) Control 24/66 (36%) NS One death due to arrest in Digoxin group	Only 56.1% of patients underwent lung resection No Holter monitoring No sample size calculation
	Ritchie et al. 1992 [67]	Unblinded PRCT	Digoxin, *n* = 58 Control, *n* = 53	Incidence of arrhythmia Digoxin 29/58 (50%) Control 19/53 (36%) NS	No Holter monitoring
				One death due to arrest in Digoxin group	

Flecainide	Borgeat et al. 1989 [71]	PRCT	Flecainide, *n* = 14 Placebo, *n* = 16	Outcome defined as need to start or increase antiarrhythmic drug Flecainide 0/14 Placebo 6/16 (38%) significant	Holter monitoring small study
	Borgeat et al. 1991 [69]	PRCT	Flecainide, *n* = 15 Digoxin, *n* = 15	SVT or complex ventricular arrhythmia Flecainide 1/15 (7%) Digoxin 7/15 (47%) significant	Holter monitoring, small study No placebo arm

Verapamil (see also study [74])	van Mieghem et al., 1996 [73]	Unblinded PRCT	Verapamil, *n* = 100 Control, *n* = 99	Incidence of AF Verapamil 8/100 (8%) Control 15/99 (15%) NS	No Holter monitoring
				23% bradycardia or hypotension in Verapamil group	

Diliazem	Amar et al. 1997 [70]	Unblinded PRCT	Diltiazem, *n* = 35 Digoxin, *n* = 35	Incidence of AF Diltiazem 5/35 (14%) Digoxin 11/35 (31%) NS	Low Diltiazem dose, No placebo arm
				Hypotension = 2 pts in Diltiazem group 2nd degree heart block = 1 in Digoxin group	
	Amar et al. 2000 [18]	Double-blinded PRCT	Diltiazem, *n* = 167 Control, *n* = 163	Incidence of AF Diltiazem 25/167 (14%) Control 40/163 (26%) significant	Good study design, Holter monitoring, sample size calculation, comparable surgical interventions, power analysis
				3.59% in Diltiazem group developed hypotension (0.61% in control)	

Amiodarone	van Mieghem et al. 1994 [74]	Retrospective review of prospectively collected data	Amiodarone, *n* = 55 No Amiodarone, *n* = 497	Incidence of AF Amiodarone 1/32 (3.1%) Verapamil 0/32 (0.0%) Control 7/32 (21.8%) (RCT report)	Study stopped early due to complications High dose of Amiodarone No Holter monitoring
				3/32 pts developed ARDS and 2/32 died in Amiodarone group (RCT report)	
				6/55 (11%) in Amiodarone group versus 9/497 (1.8%) in control developed ARDS (cohort report)	
	Lanza et al. 2003 [75]	Retrospective cohort study	Amiodarone, *n* = 31 control, *n* = 52	Incidence of AF Amiodarone 3/31 (9.7%) Control 17/52 (33%) Significant	No randomization No Holter monitoring Incidence of AF from records Low dose of Amiodarone
	Tisdale et al. 2009 [76]	PRCT	Amiodarone, *n* = 65 control, *n* = 65	Incidence of AF Amiodarone 9/65 (13.8%) Control 21/65 (32.3%) Significant	No Holter monitoring
				Shorter ICU stay for Amiodarone group pts No difference in complications	
	Riber et al. 2012 [77]	Double-blinded- PRCT	Amiodarone, *n* = 122 Control, *n* = 120	Incidence of AF Amiodarone 11/122 (9%) Control 38/120 (31.6%) significant No side effects traced to the prophylactic regime	Electrocardiogram or Holter monitoring, power analysis, intention to treat analysis

Magnesium	Terzi et al. 1996 [82]	Unblinded PRCT	Magnesium, *n* = 93 Control, *n* = 101	Incidence of atrial tachyarrhythmia Magnesium 10/93 (11%) control 27/101 (27%) Significant	No Holter monitoring No power analysis Patients in control group received Digoxin
